# The Effect of Citrus Peel Extracts on Cytokines Levels and T Regulatory Cells in Acute Liver Injury

**DOI:** 10.1155/2014/127879

**Published:** 2014-07-13

**Authors:** Ia Pantsulaia, Manana Iobadze, Nato Pantsulaia, Tinatin Chikovani

**Affiliations:** Institute of Medical Biotechnology, Tbilisi State Medical University, Chiaureli Street 2a, 0159 Tbilisi, Georgia

## Abstract

*Background*. T cell-mediated immune responses contribute to the hepatocellular injury during autoimmune hepatitis, viral infection, and hepatotoxins. Pharmacological compounds regulating immune responses are suitable candidates for prevention/treatment of this pathology. Therefore, the main aim of this study was to define the effects of antioxidant, anti-inflammatory mixture of citrus peel extract (CPE) on the immune-mediated liver injury. *Methods*. The influence of CPE on liver injury was determined by the activity of transaminases in plasma and the histological changes. Anti-inflammatory and antioxidant effects were studied by measuring frequency of T regulatory cells (Tregs), cytokines (TNF-*α*, IL-10, and IFN-*γ*), and nitric oxide levels. *Results*. The CPE application notably prevents development of liver injury through decreasing levels of both cytokines (TNF-alpha, INF) and regulatory T cells and increasing levels of IL-10. CPE injection also diminished the serum NO, which in turn resulted in evident reduction of the liver damage. *Conclusion*. Our findings represent the primary preclinical data indicating that the CPE *in vivo* could ameliorate Con A induced hepatitis. The low dose of CPE most likely can be used for the treatment of the T cell-mediated liver injury as in autoimmune hepatitis, alcoholic hepatitis, and chronic viral hepatitis.

## 1. Introduction

Despite remarkable advances of modern medicine, liver diseases caused by hepatitis virus infection, toxic insult, autoimmunity, drugs, or ischemia/reperfusion remain a severe global health problem worldwide. Irrespective of the cause, hepatic disorders are multifaceted and involve activation and infiltration of T lymphocytes in liver parenchyma causing damage there [1–5]. Compounds regulating an immune response are suitable candidates for the prevention and treatment of this pathology. However, new drug development requires studies on proper animal models representing the human liver disorders.

Concanavalin A (Con A) induced hepatitis is an ideal animal model to study T cell-mediated hepatic injury and is designed for validation of various aspects of human T cell-mediated liver diseases studies [6]. Liver damage in this model depends on CD4+CD25+ Tregs, Th17, CD4+T cells, natural killer T (NKT) cells, and Kupffer cells (KC) [6, 7]. The Th1 cytokines such as a tumor necrosis factor (TNF-*α*), interferon gamma (IFN-*γ*), interleukin-12 (IL-12), and IL-18 are key factors for the development of this disease as opposed to IL-10 that displays protective effects [6, 8]. Wei et al. showed that CD4+CD25+ Tregs play an important role in the attenuation of liver injury in Con A induced hepatitis via a TGF-*β*-dependent mechanism [9]. The mechanism of immunosuppression and potential involvement of regulatory T cells has not yet been elucidated. Particularly, two types of T cells, CD4+CD25+ FoxP3+ Tregs and NKT cells, are assumed as the main players. Tregs control autoreactive T cells* in vivo*. Reduction in their numbers or their dysfunction causes various diseases in animals and humans [10, 11]. In acute infectious diseases, Tregs inhibit immune responses to pathogens limiting collateral tissue damage. On the other hand, Tregs also cause chronic infection by overinhibiting pathogen-specific immune responses [12, 13]. Tregs have been shown to inhibit the HBV-specific T cell responses* in vitro*. Other studies demonstrated increased levels of Tregs in patients with chronic hepatitis B [14, 15]. Furthermore, Xu et al. [16] have determined that in acute hepatitis B patients the level of circulating CD4+CD25+ Tregs was initially low but in the convalescent phase the number of circulating Tregs increased and returned to the normal levels upon resolution. This makes the role of Tregs during the chronic viral infection conspicuous and can serve as a potential target for therapeutic interventions. Alternatively, modulation of the number and activity of CD4+CD25+ T cells is an obvious goal for treatment and prevention for various infections, autoimmune and tumor diseases.

Beneficial effects of citrus peel extract (CPE) on human health have recently attracted great attention. It was shown that CPE is capable of inducing programmed cell death in stomach cancer cells [17] and playing a role in the prevention of the precancerous and cancer lesions in the colon [18, 19]. The peel of citrus fruits is a rich source of flavanones and polymethoxylated flavones (PMFs) that rarely occur in other plants [20]. Several studies suggest that PMFs exhibit anti-inflammatory, anticarcinogenic, antiviral, antioxidant, antithrombogenic, and antiatherogenic effects [21, 22]. PMFs have been shown to inhibit tumor necrosis factor (TNF-*α*) and reduce the invasiveness of tumors in mice. It may also induce the differentiation of myeloid leukemic cells and promote apoptosis [21, 23]. Several groups have reported that PMFs have a positive influence on inflammatory diseases such as adjuvant arthritis in rats and toxin-induced liver injury in mice [24, 25]. In addition, the PMFs exhibit strong ability to infiltrate the small intestine and are readily absorbed into the blood circulation of the human body.

We studied the frequency of Tregs and cytokines (IL-10, IFN-*γ*, and TNF-*α*) production during Con A induced hepatitis and characterized therapeutic effects of citrus peel extract on liver injury. Our results suggest that a single injection of CPE may protect the liver from Con A induced acute injury by influencing levels of cytokines (TNF-*α*, IFN-*γ*, and IL-10) and regulatory T cells.

## 2. Materials and Methods

### 2.1. Animals

Four- to six-week-old Balb/c mice (90 in total) were obtained from the Experimental Animal Center of Tbilisi State Medical University (Georgia). Animals were fed the pellet food and water in plastic cages at 20 ± 2°C and kept on a 12 h light/dark cycle. The study complies with the current ethical regulations for animal research and is approved by the ethical committee of Institute of Medical Biotechnology at Tbilisi State Medical University.

### 2.2. Preparation of Citrus Peel Extract

Good quality citrus fruits (*Citrus sinensis* (L.) Osb.) were peeled off mechanically. The peels were dried in the shade and then ground into fine powder by pulverization. The dried powder was extracted in 12 volumes of hot water. The obtained extract was passed in chromatographic column of polyamide; the column was washed using 80% ethanol. The aqueous suspension was evaporated under vacuum at 40°C and stored for use.

### 2.3. Induction of Con A Induced Hepatitis and CPE Administration

Ninety mice were divided into 6 experimental groups by 15 animals. Concanavalin A (Sigma-Aldrich, Germany) was dissolved in sterile PBS at a concentration of 2.5 mg/mL and then used intraperitoneally (I.P) at a dose of 12.5 mg/kg of body weight (in 0.1 mL of PBS). CPE was dissolved in PBS and administered I.P. (150 [high] or 75 [low] mg/kg doses) in 30 min after Con A injection or in healthy mice. Healthy mice (control) were given the same volume of PBS. Animals were randomly divided into six groups: control (healthy), Con A (Con A treated), high/low dose of CPE (control + high/low dose of CPE), and Con A + high/low dose of CPE. Mice were anesthetized lethally at 8, 24, and 48 hours (5 mice at each time point). The plasma blood was withdrawn using a heparinized tube.

### 2.4. Analysis of Transaminase Activities

Activities of the two liver enzymes—aspartate transaminase (AST) and alanine transaminase (ALT)—in the plasma were measured by spectrophotometric method using commercially available assay kits (DiaSys Diagnostic Systems GmbH & Co. KG, Germany).

### 2.5. Detection of Nitric Oxide

A Griess assay was used to determine presence of nitric oxide. In this assay, nitrite, an end product of nitric oxide oxidation, was measured in plasma. The assay reagents included 1% (w/v) sulfanilamide (Sigma-Aldrich Inc., MO) and 0.1% (w/v) naphthylenediamine dihydrochloride (Sigma-Aldrich Inc., MO). Both reagents were dissolved in 2.5% phosphoric acid. Briefly after collecting the plasma, 50 *μ*L of both reagents was added to an equal volume of the plasma in a 96-well round bottom plate. Readings were taken in 5–10 min on an optical density plate reader at 550 nm (Labsystems, Finland).

### 2.6. Cytokine Measurement

Plasma from individual mice was collected at 8-, 24-, and 48-hour time points after Con A injection and stored below −20°C. TNF-*α*, IFN-*γ*, and IL-10 levels (pg/mL) were determined using eBiosciences ELISA assay (eBiosciences, San Diego, CA, USA).

### 2.7. Cell Preparation and Flow Cytometric Analysis

Spleen was flushed with ice-cold PBS, crushed using a tissue homogenizer, and passed through sterile mesh (70 *μ*m). Cell suspension was washed once with PBS, layered over 15 mL of Histopaque 1083 (Sigma-Aldrich), and centrifuged at 2000 rpm for 15 min at room temperature. Cells at the interface were transferred to a fresh tube and washed twice with PBS. Contaminating red blood cells were lyzed using RBC-lysis solution (eBioscience, San Diego, CA, USA). For fluorescence-activated cell sorting analysis, cells were blocked using mouse Fc-block (anti-CD16/CD32) and stained for various cell surface markers using fluorescently labeled mAbs: fluorescein isothiocyanate (FITC), phycoerythrin (PE) labeled anti-mouse CD25 (clone: PC61.5), anti-mouse-CD4 (clone: GK1.5), and anti-mouse-FOXP3 (clone: 150D/E4, eBioscience, San Diego, CA). After washing, stained cells were analyzed using a flow cytometer (FACSCalibur, BD, USA). Only live cells were counted by setting gates on forward and side scatters to exclude debris and dead cells. Isotype antibody-treated cells served as staining controls.

In order to detect intracellular FOXP3, we stained cells with surface markers (CD4 or CD25). Splenocytes were fixed and permeabilized using the cytofix/cytoperm kit according to the manufacturer's instructions (BD Biosciences) and stained using anti-mouse-FOXP3 (clone: 150D/E4, eBiosciences, San Diego, USA). Fluorescent assessments were carried out on FACSCalibur (BD Biosciences, USA). Thresholds for positive phenotypic expression were delineated with the respective isotype controls using WinMidi version 2.9 software (Scripps Inst., La Jolla, CA, USA).

### 2.8. Statistical Analyses

Statistical analyses were done using Statistica 8.0 (Statsoft, Minneapolis, USA). Data express the mean + SE (standard error). The statistical significance between treatment and control groups was determined by factorial ANOVA. The data of liver histological changes were analyzed using nonparametric test. *P* < 0.05 was considered significant.

## 3. Results

### 3.1. CPE Influence on the Con A Induced Liver Injury

**Figure 1:**
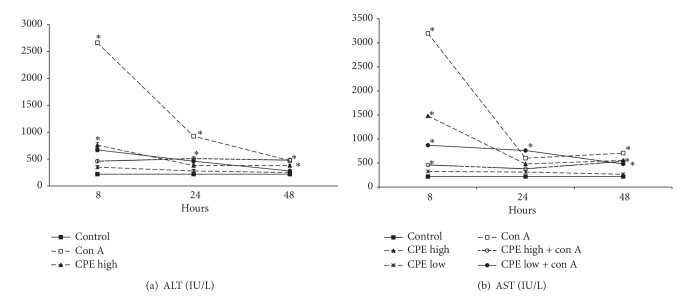
Hepatic injury after Con A administration and/or CPE treatment in mice. Con A (12.5 mg/kg body weight) was administered intraperitoneally to Balb/c mice. Serum ALT (a) and AST (b) levels (mean ± SE) were measured at 8, 24, and 48 hours (5 mice at each time point) after Con A injection. *P < 0.05 (compared with control group).

To examine whether CPE treatment had protective effects on the Con A induced liver injury, two different doses (150 mg/kg, 75 mg/kg) of CPE were administered intraperitoneally to mice. The Con A injected animals developed acute hepatitis as indicated by elevated serum AST and ALT levels (Figures 1(a) and 1(b)). Liver enzyme levels peaked at 8-hour postinjection, and later at 24-hour time point they started to subside (*P* < 0.05). CPE treatment had a protective effect on the Con A induced liver injury in a dose-dependent manner. Mice treated with CPE immediately after the Con A challenge showed a significant slant in AST and ALT curves at 8-, 24-, and 48-hour posttreatment (Figures 1(a) and 1(b)). Treatment with a low dose (75 mg/kg) of CPE markedly protected mice from the Con A induced ALT and AST elevated levels in serum (*P* < 0.05), whereas a high dose (150 mg/kg) of CPE did not incur inhibition, perhaps attributable to side/toxic effects.

### 3.2. CPE Enabled Protection of the Con A Induced Hepatitis by Suppression of Cytokines

**Figure 2:**
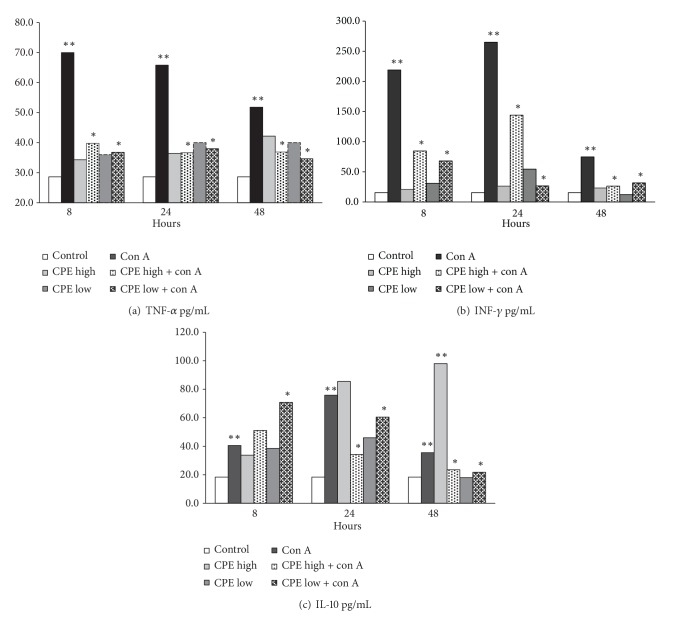
Circulatory cytokines ((a) TNF-*α*, (b) INF-*γ*, and (c) IL-10) levels (mean ± SD, pg/mL) in all studied groups at 8, 24, and 48 hours after Con A injection (5 mice at each time point). *P* < 0.05 (**compared with control, *compared to Con A treated mice).

Cytokines are key regulatory molecules of the immune system. TNF-*α*, interleukin-6 (IL-6), and interleukin-10 (IL-10) play an important role in mediation of inflammatory processes. The Con A induced hepatitis is associated with production of various cytokines. We evaluated kinetics of TNF-*α*, IFN-*γ*, and IL-10 levels during the Con A induced liver injury (Figure 2). In the Con A group, the serum level of TNF-*α* reached its peak at the 8-hour time point after the Con A injection (Figure 2(a), *P* < 0.05). Both, high and low, doses of CPE greatly inhibited induction of TNF-*α* at the 8-hour time point by Con A (Figure 2(a), *P* < 0.05). In contrast to the rapid surge of TNF-*α*, the level of IFN-*γ* peaked at 24 hr after Con A injection, while a low dose of CPE significantly decreased IFN-*γ* levels at 24 hr (Figure 2(b), *P* < 0.05). Injection of a high dose of CPE caused elevation of the IFN-*γ* levels that subsided to the baseline by 48 hr. In contrast, in the Con A treated mice IFN-*γ* levels did not revert to their baseline by 48 hr (Figure 2(b)).

IL-10 serum levels increased at the 8 hr and 24 hr time points of the post-Con A treatment. Compared with the control group, significantly higher levels of serum IL-10 were observed in both doses of the CPE/Con A treated mice (Figure 2(c), *P* < 0.05). The results of all time points (8, 24, and 48 h) conveyed statistical significance (Figure 2(c)).

### 3.3. CPE Effect on NO Content in Peripheral Blood of the Con A Treated Mice

**Figure 3:**
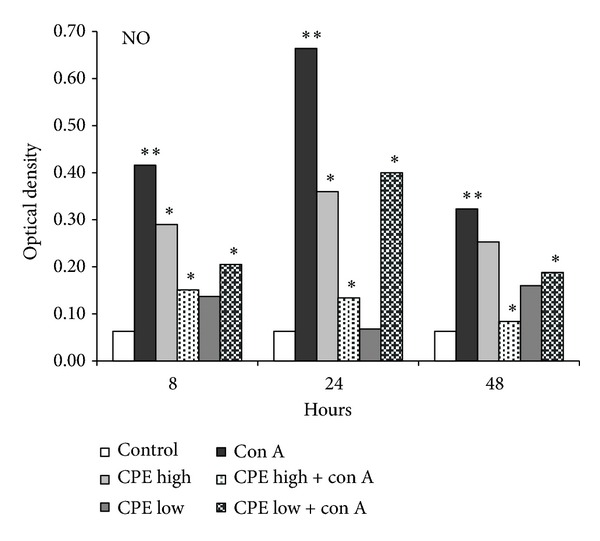
Nitric oxide levels (mean ± SE, optical density) in serum of all studied mice (6 groups, 5 mice in each time point) at 8, 24, and 48 hours* after* Con A injection + CPE treatment. *P* < 0.05 (**compared with control, *compared to Con A treated mice).

To determine whether CPE also have influence on inflammatory mediator expression, we examined its effects on NO levels in peripheral blood. After the administration of high dose of CPE, the NO content decreased by more than 60% (Figure 3, *P* < 0.05). Furthermore, the CPE-treated mice experienced halving of their plasma nitrite concentrations indicating that significantly less NO was released into plasma.

### 3.4. CPE Treatment Increases CD4+CD25+FOXP3+ Cells in Spleen of the Mice with Con A Induced Hepatitis

**Figure 4:**
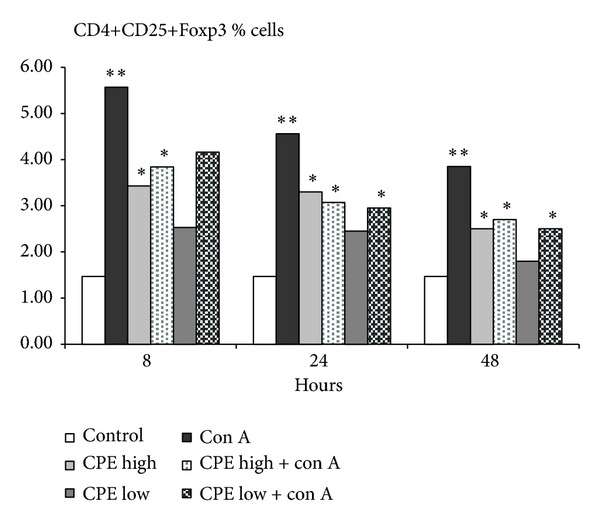
The frequency of CD4+CD25+Foxp3+ cells (percentage, mean ± SE) in case of immune-mediated liver injury and the effects of treatment with high or low doses CPE at 8, 24, and 48 hours after Con A injection (5 mice each time point). *P* < 0.05 (**compared with control, *compared to Con A treated mice).

We also measured quantities of Tregs (CD4+CD25+Foxp3+) in the Con A injected mice and studied influence of CPE treatment on these cells (Figure 4). Compared to the control group, Con A injection led to a high level of Treg population in mice spleen. The Foxp3 expression levels were significantly increased in splenic Tregs and declined at 24 hr of Con A injection (Figure 4). The levels of splenic Tregs however did not return to their baseline (Figure 4). Treatment with low and high doses of CPE forced down the percentage of CD4+CD25+Foxp3+ regulatory T cells in the Con A injected mice to a great extent (Figure 4, *P* < 0.05).

## 4. Discussion

Citrus fruit-derived flavonoids and their metabolites have various biological activities, including anti-inflammatory and antioxidant properties. The existing research evidence suggests that flavonoids extracted from citrus fruit peels are the most bioactive on Earth, even more than the citrus fruit juice sac components. The present study demonstrates that CPE has hepatoprotective and immunosuppressive effects. The results of our study show that treatment with a low dose of CPE reduced the Con A induced acute liver injury evidenced by decreased amounts of aminotransferases (Figure 1) and concurrent histomorphological changes (picture not shown).

We revealed that the CPE treatment remarkably cut down on inflammatory cytokines (TNF-*α* and IFN-*γ*) within 8 hr after the Con A injection (Figure 2, *P* < 0.05). At the same time, CPE infusion elevated IL-10 levels and displayed hepatoprotective effects leading to inhibition of TNF-*α* and IFN-*γ* production. TNF-*α*, IFN-*γ*, and IL-4 produced by macrophages, regulatory T cells, and natural killer T (NKT) cells play important roles in the development of the Con A induced hepatitis [26]. In contrast, IL-6 and IL-10 cytokines protect mice from the Con A induced acute liver injury [27]. The primary effect of either endogenous or exogenous IL-10 in case of acute liver injury is the inhibition of Th1 responses. Several investigators [8, 16] demonstrated a critical role of IL-10 in Con A tolerance [8, 16]. Our experiment shows that CPE effects are mediated by suppression of TNF-*α* and elevation of IL-10.

Tregs have been shown to be an important factor in the attenuation of liver injury [28]. Increased frequency of Tregs has been seen only in the T cell-mediated liver injury, not in any other immune cell-mediated liver injury such as NK or Kupffer cells. We examined frequency of splenic CD4+CD25+Foxp3+ Tregs and observed the increased number of Foxp3 Tregs after 8 hr. This effect started to decline at 24 hr time after the Con A injection. Our results are in agreement with reports of Erhardt and Tiegs [8]. We also observed that, compared to the Con A injected mice, Con A injection plus a high dose of CPE treatment significantly diminished content of CD4+CD25+Foxp3+ regulatory T cells (Figure 4). Note that only the mice treated with a high dose of CPE showed an insignificant surge in the number of regulatory T cells vis-à-vis the control group.

Our findings partly diverge from the results of Wei et al. [9] who showed significant increase in the absolute number of Tregs in liver after Con A injection at all times. In contrast to the increased number of Tregs in liver, there were no significant changes in the number of Tregs in spleen following Con A injection. Yet, expression levels of Foxp3, assessed as mean fluorescence intensity, were significantly increased in both hepatic and splenic Tregs but started to decline earlier in splenic Tregs [9]. This discrepancy is almost certainly attributable to using the different models (dose and times of Con A injection). We hypothesized that fluctuation in the content of CD4+CD25+Foxp3+ cells is related to changes of cytokines levels, which are strongly induced upon the first Con A challenge (IFN-*γ*, TNF-*α*, and IL-17). These cytokines probably induce generation and recruitment of specialized Tregs as previously demonstrated by other researches [29, 30].

On the other hand, the CPE has been shown to have a bearing on multiple key elements in signal transduction pathways related to the cellular proliferation, differentiation, apoptosis, inflammation, and obesity. The majority of the citrus fruit peels suppress LPS-induced NO production in RAW 264.7 cells [31]. Compared to the immature fruit peels, the inhibitory effect of mature fruit peels on NO production was significantly weaker suggesting that it can be determined by the flavonoid composition. NO mediates tissue injury through several pathways including inhibition of mitochondrial respiration, inactivation of proteinase inhibitors, and formation of free radicals. In other words, NO is a substance extremely toxic to cells. The CPE treatment significantly decreased the serum NO content, which in turn resulted in evident reduction of the liver damage, compared to the control group. Our results suggest that hepatoprotective effects of CPE consist in the ameliorating faculty of the endogenous antioxidant system.

After all, we have certain limitations in this study. First, we did not examine direct interaction of Tregs with cytokines in liver. Second, depletion of IL-10 or IFN-*γ* was not investigated with specific anti-cytokine antibodies, which could have given us extra insights into the mechanism of CPE effects in the T cell-mediated liver injury. Nevertheless, our work provides a new understanding of a CPE therapeutic effect during the Con A induced hepatic injury.

In conclusion, our findings offer sound evidence that application of both high and low doses of CPE exhibits protective effects on T cell-dependent hepatitis, possibly through suppression of inflammatory mediators. However, treatment with only high dose of CPE has side effects on the liver. Our results come into agreement with Gosslaua et al. [32] findings. This study indicates that the high dose of orange peel extract, enriched with different concentration of polymethoxyflavones (PMFs), has strong anti-inflammatory effect but due to the increased concentration of OH-PMFs the cytotoxicity is high as well. Based on our results and existing literature summary [32–34], we can assume that the toxic effects of high dose CPE are most likely associated with the high concentration of PMFs.

The low dose of CPE can most likely be used for the treatment of the T cell-mediated liver injury as in autoimmune hepatitis, alcoholic hepatitis, and chronic viral hepatitis. Our results point to CPE as a potential substance against the immune-mediated hepatitis. Future mechanistic studies in mice and in humans are capable of providing more detailed information on protective effect of CPE and help to establish its safety and potential use in clinics.
